# Supplementary material for: Convolutional neural networks develop major organizational principles of early visual cortex when enhanced with retinal sampling
Source: Sci Rep. 2024 Apr 18;14:8980. doi: 10.1038/s41598-024-59376-x (PMC11026486; doi:10.1038/s41598-024-59376-x)
Supplement: Supplementary file 1 — Supplementary Information. [file 41598_2024_59376_MOESM1_ESM.pdf]

Convolutional Neural Networks Develop Major Organizational Principles of Early Visual Cortex when Enhanced with Retinal Sampling

Supplementary Materials

Danny da Costa*, Lukas Kornemann, Rainer Goebel and Mario Senden

*Corresponding author(s). E-mail(s): danny.dacosta@maastrichtuniversity.nl;

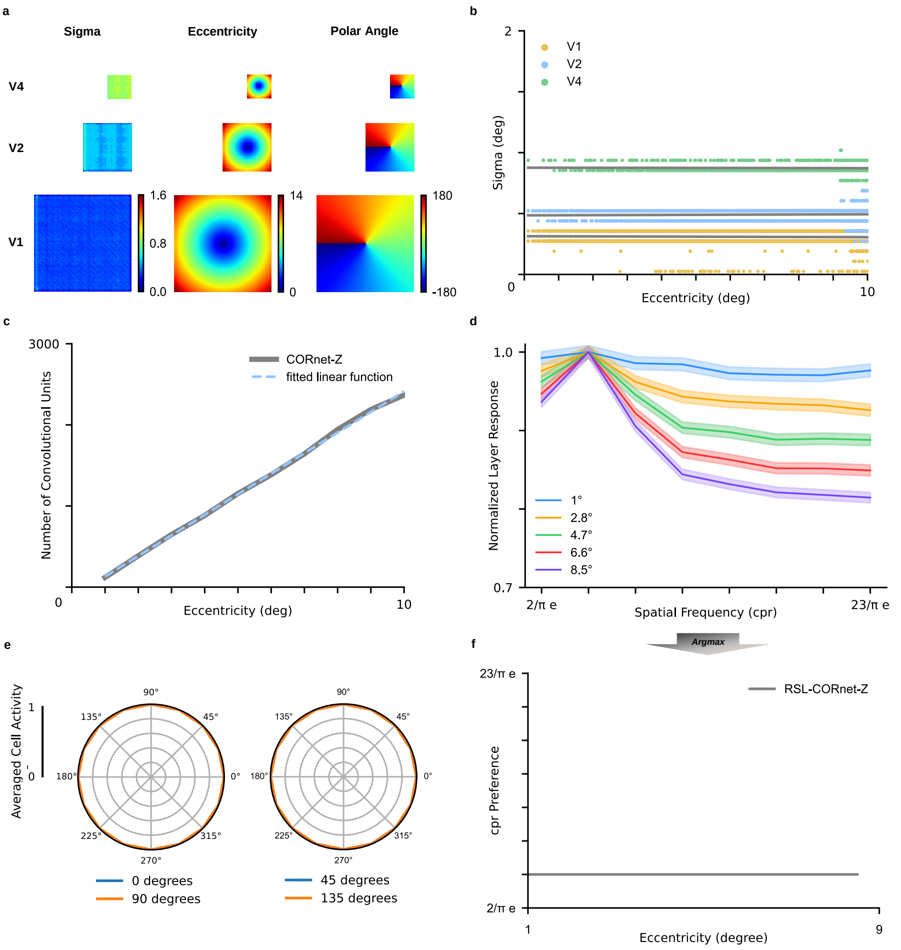

Supplementary Figure 1 Results for standard CORnet-Z. **a**, Retinotopic maps of convolutional layers. **b**, Receptive field size as a function of eccentricity for V1, V2, and V4 layers. **c**, Convolutional units per level of eccentricity for the V1 layer. **d**, Normalized averaged layer response for the V1 layer as a function of spatial frequency. **e**, Polar plots displaying radial bias for 4 c/deg sinusoidal gratings. The left plot shows the result for 0° vs 90° sinusoidal gratings, the right plot shows 45° vs 135°. **f** Spatial frequency preference as a function of eccentricity.

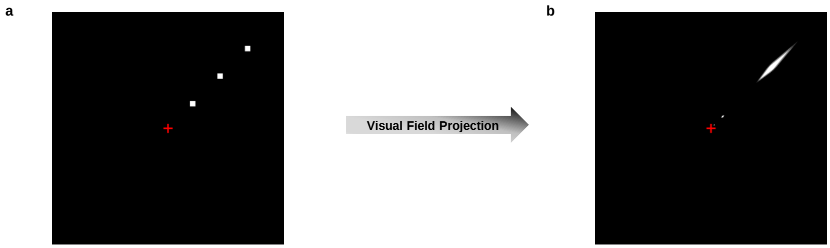

Supplementary Figure 2 Visualization of receptive fields. **a**, Visualization of V1 receptive fields. Sampling is performed on the output of the RSL layer. **b**, Identical receptive fields as in a, but projected in the visual field. Receptive fields are elongated towards the fovea. The amount of elongation is a function of eccentricity.

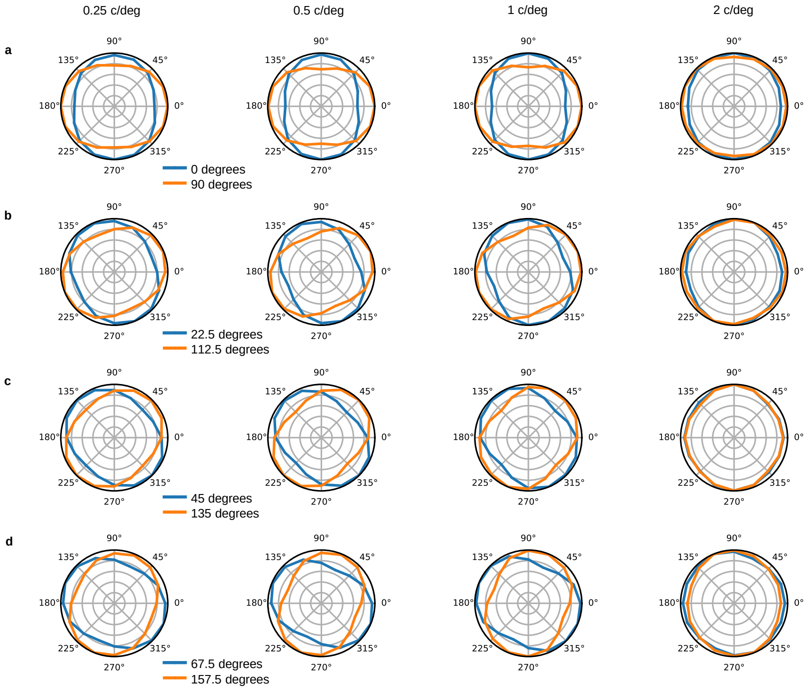

Supplementary Figure 3 RSL-CORnet-Z radial bias polar plots for lower spatial frequencies. The columns show the results for 0.25, 0.50, 1, and 2 c/deg gratings, respectively. **a**, Polar plots show the results for 0° vs 90° sinusoidal gratings. **b**, Results for 22.50° vs 112.50° gratings. **c**, Polar plots for 45° vs 135° gratings. **d**, Results for 67.5° vs 157.5° gratings.

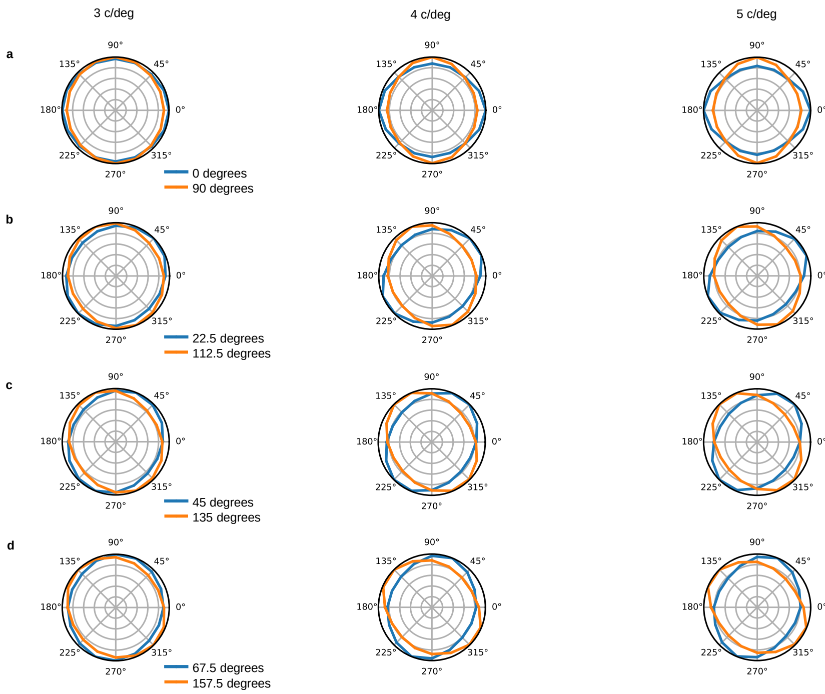

Supplementary Figure 4 RSL-CORnet-Z radial bias polar plots for the higher spatial frequencies. The columns show the results for 3, 4, and 5 c/deg gratings, respectively. **a**, Polar plots show the results for 0° vs 90° sinusoidal gratings. **b**, Results for 22.50° vs 112.50° gratings. **c**, Polar plots for 45° vs 135° gratings. **d**, Results for 67.5° vs 157.5° gratings.

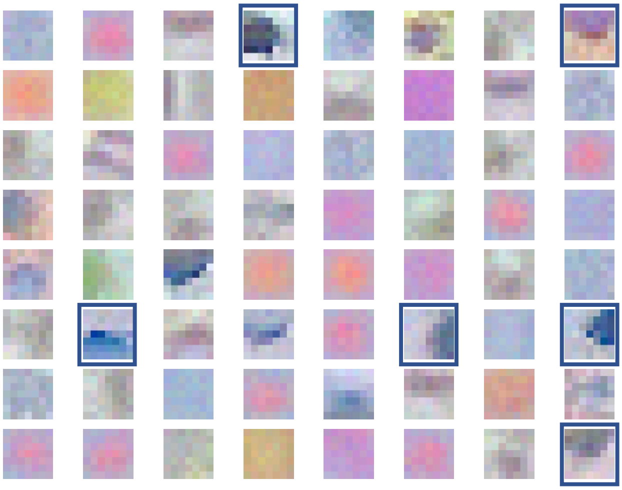

Supplementary Figure 5 Visualization of V1 filters for RSL-CORnet-Z. V1 convolutional filters exhibit a curved profile to compensate for non-uniform distortion in visual input. Some filters show a notable curvature, which is highlighted by blue boxes in the image. Note that the extent of filter curvature is subjective.
